# Therapeutic Effect and Metabolic Mechanism of A Selenium-Polysaccharide from *Ziyang* Green Tea on Chronic Fatigue Syndrome

**DOI:** 10.3390/polym10111269

**Published:** 2018-11-15

**Authors:** Changzhuan Shao, Jing Song, Shanguang Zhao, Hongke Jiang, Baoping Wang, Aiping Chi

**Affiliations:** 1 College of Arts and Sciences, Shanghai Maritime University, Shanghai 201306, China; czshao@shmtu.edu.cn (C.S.); hkjiang@shmtu.edu.cn (H.J.); 2 Laboratory of Nutrition and Hygiene, Shaanxi Normal University, Xi’an 710119, China; BSUjingjing@163.com (J.S.); zhaoshan@snnu.edu.cn (S.Z.); wbp1979@snnu.edu.cn (B.W.)

**Keywords:** polysaccharide, chronic fatigue syndrome, metabonomics

## Abstract

*Ziyang* green tea was considered a medicine food homology plant to improve chronic fatigue Ssyndrome (CFS) in China. The aim of this research was to study the therapeutic effect of selenium-polysaccharides (Se-TP) from *Ziyang* green tea on CFS and explore its metabolic mechanism. A CFS-rats model was established in the present research and Se-TP was administrated to evaluate the therapeutic effect on CFS. Some serum metabolites including blood urea nitrogen (BUN), blood lactate acid (BLA), corticosterone (CORT), and aldosterone (ALD) were checked. Urine metabolites were analyzed via gas chromatography-mass spectrometry (GC-MS). Multivariate statistical analysis was also used to check the data. The results selected biomarkers that were entered into the MetPA database to analyze their corresponding metabolic pathways. The results demonstrated that Se-TP markedly improved the level of BUN and CORT in CFS rats. A total of eight differential metabolites were detected in GC-MS analysis, which were benzoic acid, itaconic acid, glutaric acid, 4-acetamidobutyric acid, creatine, 2-hydroxy-3-isopropylbutanedioic acid, l-dopa, and 21-hydroxypregnenolone. These differential metabolites were entered into the MetPA database to search for the corresponding metabolic pathways and three related metabolic pathways were screened out. The first pathway was steroid hormone biosynthesis. The second was tyrosine metabolism, and the third was arginine-proline metabolism. The 21-hydroxypregnenolone level of rats in the CFS group markedly increased after the Se-TP administration. In conclusion, Se-TP treatments on CFS rats improved their condition. Its metabolic mechanism was closely related to that which regulates the steroid hormone biosynthesis.

## 1. Introduction

The typical presentation of chronic fatigue syndromes (CFS) are characterized by sudden onset of a flu-like illness. Its predominant symptoms is severe long-lasting fatigue that greatly affect activity and bodily condition [1]. There are pensive changes in patients’ physical, mental, and occupational state. They may face social isolation for some time [2]. The commonly used criterion is from the U.S. Centers for Disease Control and Prevention (CDC), which refers to the assessment system of Holmes and Fukuda [3]. Among developed countries, the prevalence rate is between 0.2% and 2.5% [4,5]. Moreover, the rate is 1.9% in Beijing and the prevalence at Hong Kong is 3% [6]. Some hypotheses consider CFS’s pathogenesis to be related to excessive free radical generation, hypothalamic–pituitary–adrenal (HPA) axis disorder, and immune dysfunction. A recent study demonstrated that there was a higher oxidative stress index and more reactive oxygen metabolites-derived compounds in patients with CFS [7]. CFS attenuates the diurnal variation of cortisol and causes a blunted HPA axis responsiveness [8]. Moreover, many CFS patients had higher anti-inflammatory cytokines, abnormal autoantibodies, and immune complex allergies [9].

However, there were not many effective therapies in conventional medicine. Some anti-fatigue foods or natural compounds without side effects were chosen to treat CFS [10,11]. Polysaccharides are a kind of active components in plants with adjusting immune function and enlarging antioxidant capacity [12,13,14]. Furthermore, it has been reported that the polysaccharides could treat CFS [15]. It was also known that selenium (Se) was not only an essential element for human body but also an active center of glutathione peroxidase [16,17]. Moreover, selenium-enriched polysaccharides have been demonstrated to reflect stronger biological activities than normal polysaccharide [18,19]. As a consequence, exploring the beneficial efficacy of selenium-enriched polysaccharides for health has become a hot field. *Ziyang* green tea grows widely in Ziyang County, which is famous for its selenium-enriched soil in China. Previous research reported that the selenium-enriched polysaccharides from *Ziyang* green tea displayed various biological activities, such as anti-aging, antioxidant, and immunological enhancement [20,21,22]. Our previous research discovered that the selenium-enriched polysaccharides from *Ziyang* green tea improved the exercise-induced fatigue of rats [23].

Metabolomics study on all sets of molecular compounds, whether they are familiar to people or stay unknown, rather than work on individual metabolites separately, giving us a prospect on the metabolic function of body [24]. The therapeutic mechanism and effect of the biological active substances in the body can be evaluated through the method of metabonomics. In particular, studying the differences of metabolites after the treatment of medicine can be helpful to diagnose disease and find biomarkers. Our previous report showed that combined gas chromatography-mass spectrometry (GC-MS) was effective in finding biomarkers through samples while considering the CFS state [25]. 

In the present research, we studied physicochemical properties of the selenium-enriched polysaccharide (Se-TP) from *Ziyang* green tea, and then evaluated the therapeutic effect of Se-TP on CFS through a rat model. The GC-MS method in metabonomics and multivariate statistical programs were used to measure and analyze the data to discover some differential metabolites of CFS, which assisted in figuring out biomarkers associated with CFS and the explored metabolic mechanism of Se-TP against CFS.

## 2. Materials and Methods 

### 2.1. Materials and Reagents

*Ziyang* green tea was obtained from Ziyang Selenium-Rich Food Co., Ltd. (Ziyang county, China) and identified as a specific variety of selenium-enriched C. *sinensis L*. by botanist Prof. Y. Ren (College of Life Science, Shaanxi Normal University, Xi’an, China). The chemicals were of analytical reagent grade and were purchased from Sinopharm Chemical Reagent Co., Ltd. (Shanghai, China).

### 2.2. Physicochemical Characteristic of Se-TP

The extraction, separation, and characterization of Se-TP were performed as in our previous report [23]. Briefly, the polysaccharide content was determined using the anthrone-sulfuric acid method and its monosaccharide compositions were detected using a high-performance liquid chromatography (HPLC) system (LC-2010A, Shimadzu, Kyoto, Japan). The binding protein and amino acid content were measured with an amino acid analyzer (Hitachi L-8900, Tokyo, Japan). Se content was measured using graphite furnace atomic absorption spectroscopy (ZA3000, Hitachi, Tokyo, Japan), and the molecular morphology was observed using a scanning electron microscope (SEM) (Quanta 200, Philips–FEI Company, Amsterdam, Holland).

### 2.3. Establishment of CFS Model

**Figure 1:**
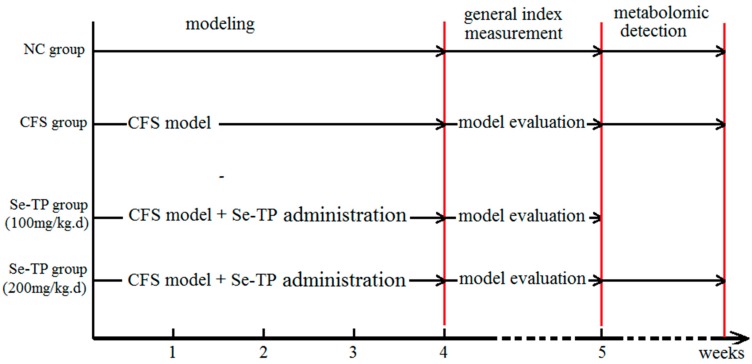
Schedule of experimental design.

The CFS model was established as we described previously [15]. Forty SD rats (140–160 g, 5–6 weeks old) were purchased from the Experimental Animal Center of Xi’an Jiaotong University (Xi’an, China) (License No.: SCXK(SHAN)2012-003). Animals were acclimated for 7 days, and then divided into four groups: normal control (NC), CFS, and two Se-TP treatment (100 and 200 mg/kg) groups, and each group had 10 rats. According to some research [26,27,28], this study mimics the multi-factor sick reason of CFS, which adopted the following features: restraint stress, physical fatigue, and crowded environments. The rats in the CFS and the Se-TP treatment groups were administrated in the above conditions for four weeks in total. Restraint-stress experiments were performed using our patent instruments (patent number: ZL201620136043.7, China’s State Intellectual Property Office), in which the rat was fixed for 4 h each day. Then, exercise on a treadmill (25 m/min) for 1 h to reach physical fatigue was used. Furthermore, 10 rats were housed crowded and noisy together in a rearing cage and exposed to music for 12 h a day. Se-TP was dissolved in a small amount of saline. The rats in the Se-TP groups were given Se-TP solution through an oral gavage for 4 weeks, and the doses were 100 and 200 mg/kg/day, respectively. The rats in the NC and CFS groups were given the same volume of saline. The weight and dietary intake of the rats were recorded after 4 weeks. The Morris water maze test, the open-field test, and the tail-suspension test were carried out to assess the CFS model [15]. Briefly, the time of searching the safe platform was recorded, the times of crossing through the adjacent grids, and the times of standing were recorded separately. In tail-suspension test the motionless time was recorded. The schedule of experimental design is shown in Figure 1. This study was reviewed and approved by the Special Committee on Scientific Ethics of Shaanxi Normal University.

### 2.4. Analysis of Serum Metabolites

Rats were anesthetized with 25% urethane (i.p., 0.5 mL/100 g) and their blood was drawn to prepare a serum via centrifugation at 1610× *g* at 4 °C for 10 min. Levels of blood urea nitrogen (BUN) and blood lactate acid (BLA) in serum were determined using the biochemical analyzer (Hitachi 7020, Hitachi, Tokyo, Japan). Corticosterone (CORT) and aldosterone (ALD) was measured using commercial ELISA kit (Cayman Chemical Company, Ann Arbor, MI, USA). 

### 2.5. Urine Sample Preparation and Metabolites Extraction

After the behavior test and the serum metabolites analysis, only Se-TP (200 mg/kg) samples with better therapeutic effect were used for the metabolomics study. Therefore, those rats in NC, CFS, and Se-TP (200 mg/kg) groups were housed in the metabolic cages (our utility patents, patent number: ZL-201620136043.7) for 6 h to collect urine. The urine samples were collected and stored at −80 °C to await GC-MS analysis. 

Urine samples were thawed and centrifuged at 15,600× *g* for 10 min. Then 100 μL of the sample suspension was added to the same volume of water and 10 μL of urease suspension to incubate at 37 °C for an hour. A total of 0.30 mL of methanol and 500 μL of acetonitrile were added, mixed, and centrifuged. Next, 400 μL of supernatant was dried and 80 μL of methoxyamine pyridine solution was added. The mixed solution was incubated and derived for GC-MS analysis.

### 2.6. GC-MS Detection

GC-MS detection was performed using a gas chromatograph system (Agilent 7890, San Francisco, CA, USA) and a mass spectrometer (Pegasus HT, Hazel Grove, UK). A Rxi-5Sil MS column (30 m × 250 μm × 0.25 μm, Restek, Bellefonte, PA, USA) was adopted in this system. Operating conditions in the GC-MS detection were as follows. Sample size: 1-μL. Front inlet purge flow: 3 mL/min. Gas flow in column: 20 mL/min. Initial temperature: maintained at 50 °C for 1 min and then increased to 330 °C for 5 min. The energy was −70 eV in the electron impact mode. The mass spectrometry data: 30–600 *m*/*z* full-scan.

### 2.7. Statistics and Analysis of Data

The body weight, dietary intake, and behavioral performance were analyzed using SPSS software (version 17.0, IBM, Armonk, NY, USA). The data are reported as the means ± standard deviations (SD). Differences among means were determined using analysis of variance (ANOVA) with Tukey’s test. Values of *p* < 0.05 and *p* < 0.01 were considered statistically significant.

The detected peaks were aligned and analyzed using manual integral methods. The data from LECO/Fiehn Metabolomics Library was used to identify the compounds, which gave similarity values for the compound identification accuracy. The similarity of the compounds exceeding 700 that could be adopted. Multivariate statistics and univariate statistics were used to evaluate the data. Data pre-processing was done as follows. In order to better analyse the downstream data, the missing values in the original data were simulated using half of minimum method, and then the data were standardized using the internal standard normalization method. An orthogonal partial least squares discriminant analysis (OPLS-DA) was done using SIMCA software (Version 14.0, Umetrics AB, Umea, Sweden) from normalized data. Furthermore, it can build a model to check the component variation of the four groups. The Kyoto Encyclopaedia of Genes and Genomes (KEGG; http://www.genome.jp/kegg/) mass spectral libraries were utilized to confirm the differential metabolites. MetaboAnalyst (http://www.metaboanalyst.ca/) was used to search for the pathways of metabolites, which is a comprehensive online tool suite for the statistical and functional analysis of metabolomics data, created by members of the Wishart Research Group at the University of Alberta, Edmonton, AB, Canada. The data of selected metabolites were entered into MetaboAnalyst. “Pathway analysis,” “Homo sapiens,” “Hypergeometric test,” and “Relative-betweenness centrality” were selected in metabolic mode, species types, characterization analysis, and path topology analysis, respectively. Then, the topological score was analyzed to screen the main changed metabolic pathways. 

## 3. Results

### 3.1. Composition and Morphology of Se-TP

**Figure 2:**
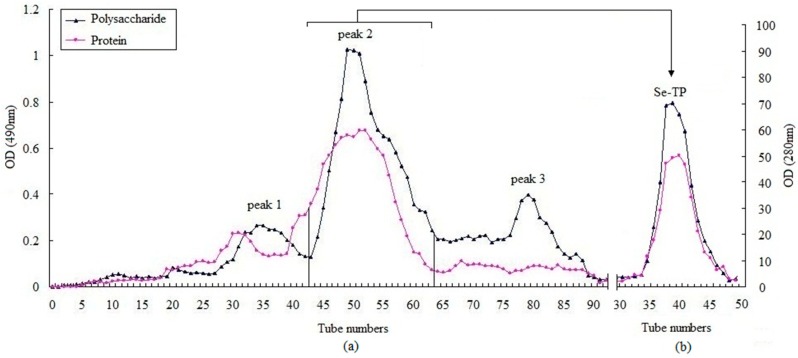
Separation and purification of Se-TP: (**a**) separation of the crude polysaccharides using a DEAE-52 cellulose column, and (**b**) purification of sample using a Sephadex G-150 column.

**Figure 3:**
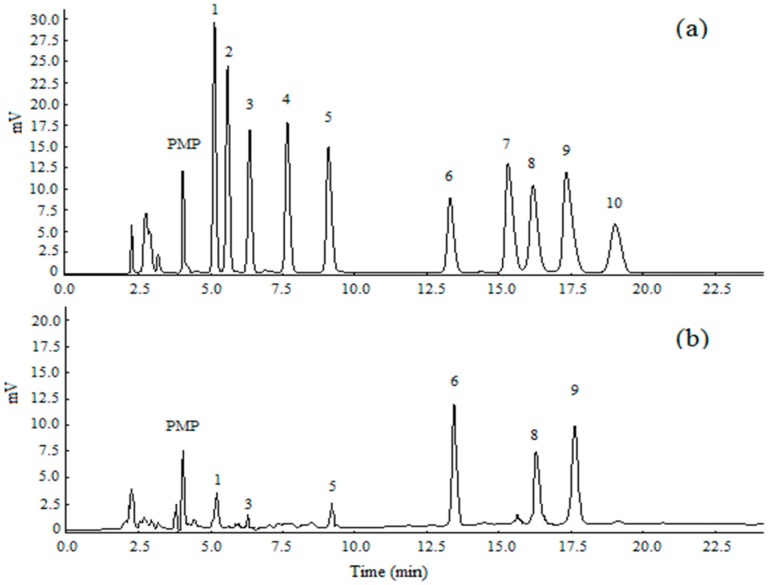
Detection results of samples using an HPLC chromatograms test: (**a**) standard monosaccharides (1—mannose, 2—ribose, 3—rhamnose, 4—glucuronic acid, 5—galacturonic acid, 6—glucose, 7—xylose, 8—galactose, 9—arabinose, 10—fucose); (**b**) monosaccharide composition of Se-TP.

**Figure 4:**
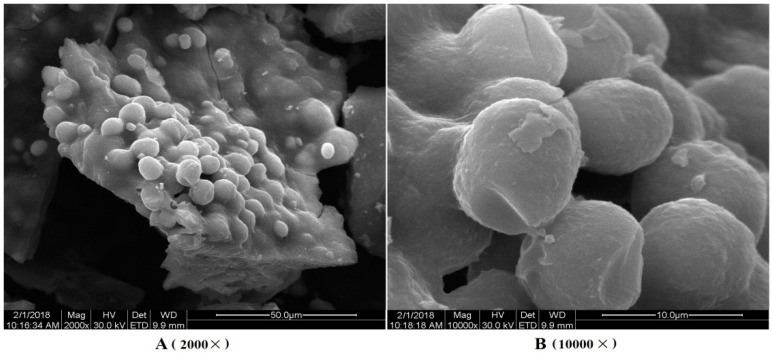
Molecular morphology images of Se-TP: (**A**) at magnifications of 2000×, and (**B**) at magnifications of 10,000×. The diameter of the spherical particles was approximately 5 μm and the surface was smooth.

The crude polysaccharides were extracted from the dried *Ziyang* green tea using hot-water extraction and ethanol precipitation, and then were separated in a DEAE-52 cellulose column (Beijing Dingguo Biotechnology Co. Ltd., Beijing, China). Three peaks were detected (Figure 2a) with yields of 12.34%, 53.69%, and 16.51%. The second peak was further purified in a Sephadex G-150 column (Beijing Dingguo Biotechnology Co. Ltd., Beijing, China). A symmetrical peak of polysaccharide was detected and the curves of protein were superposed with it (Figure 2b). The sample was collected and named Se-TP. The main ingredients of Se-TP included polysaccharide, binding protein, and Se with percentages of 83.5%, 4.58%, and 1.86%, respectively. The monosaccharide compositions were composed mainly of mannose, rhamnose, glucose, galactose, arabinose, and galacturonic acid with molar ratios of 3.57%, 1.69%, 32.35%, 25.81%, 30.64%, and 2.26%, respectively (Figure 3). The morphology of Se-TP was observed by the scanning electron microscope. As shown in Figure 4, Se-TP resembled large blocks under 2000× conditions (Figure 4A), which appeared as many spherical particles under 10,000× conditions (Figure 4B). The diameter of the spheres was approximately 5 μm, and the surface appeared to be smooth.

### 3.2. Behavioral Test Results

The behavioral test results of rats are shown in Table 1. The average amount of feed intake per day and growth rate of body weight significantly decreased in the CFS group rats when compared with those in the NC group (*p* < 0.01), which showed that the growth and development of rats were inhibited by CFS. Furthermore, the time of searching for the platform significantly increased in the Morris water maze test but the number of standing events and number of steps out of the middle grid markedly decreased in the open-field test in the CFS group versus the NC group (*p* < 0.05 or 0.01). In addition, the motionless time in tail-suspension test of CFS group rats significantly lengthened compared with those in the NC group (*p* < 0.01). The above results indicated chronic stress resulted in physical and mental fatigue in the CFS rats. 

The results of the comparison between the Se-TP (200 mg/kg) groups and the CFS group showed that the average amount of feed intake, the number of standing events, and number of steps out of the middle grid were dramatically increased, whereas the time of searching for the platform and the motionless time were significantly reduced. For the administration of 100 mg/kg of Se-TP, only the motionless time of the rats markedly decreased in comparison with the CFS group. That illustrated the high-dose of Se-TP was better in the therapeutic effect than the low-dose of Se-TP.

### 3.3. Analysis of Related Serum Metabolites

The results of the serum metabolites measurement were shown in Table 2. The BUN levels of rats in the CFS group were significantly increased in comparison with those in the NC group (*p* < 0.05), and the serum CORT contents of them were significantly lower than those in the NC group (*p* < 0.05). However, the levels of BLA and ALD did not show significant difference between both groups. In addition, the BLA level of rats in the Se-TP (200 mg/kg) group was significantly decreased, whereas the ALD level of them was markedly increased when compared with those in the CFS group (*p* < 0.05). Meanwhile, there were no significant differences between Se-TP (100 mg/kg) group and CFS group.

### 3.4. Analysis of Urine’s Metabolic Profiling

**Figure 5:**
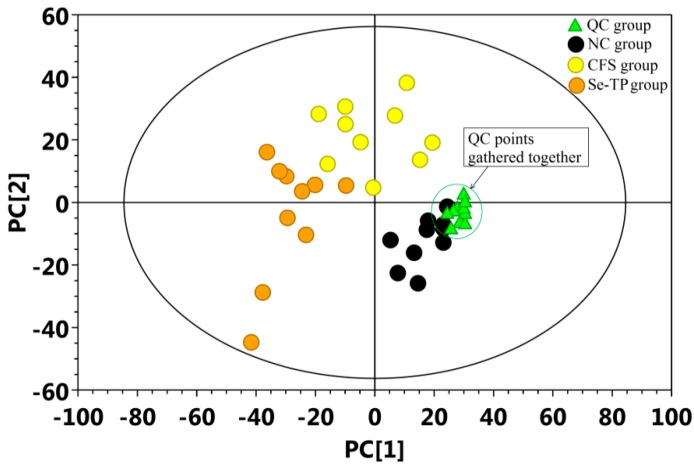
Evaluation of GC-MS detection model through the PCA scores plot of QC samples. Gathered points of QC sample showed that the PCA model was reliable.

Total ion chromatograms (TICs) of urine samples from the NC, CFS, and Se-TP groups are presented in Figure S1. A total of 328 original peaks were obtained from 30 samples and 10 quality control (QC) samples. These data were filtered using two methods: interquartile range or keeping single null values less than or equal to 50%, and then standardized using the internal normalization method. Finally, 281 peaks were chosen and inputted into SIMCA software for recognition analysis using the multivariate pattern. The gather degree of the QC sample score plot was the standard to evaluate the PCA model. The result showed that the points of QC sample were closely gathered together, which showed the PCA model was reliable (Figure 5).

**Figure 6:**
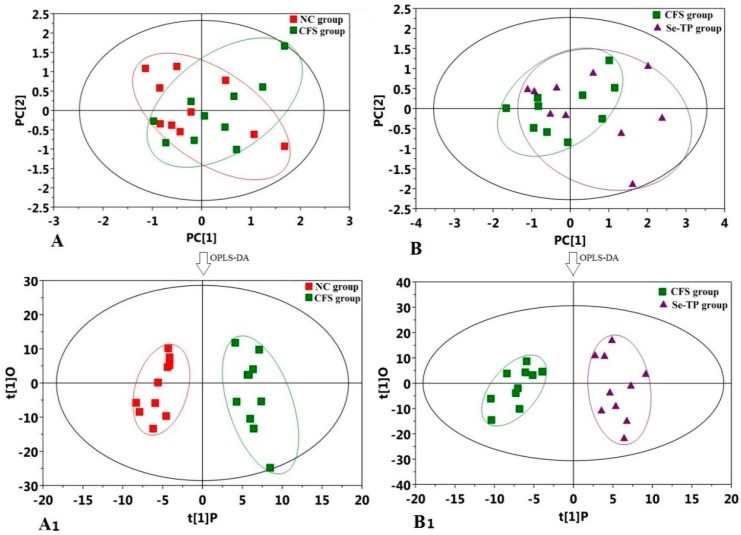
Results of multivariate statistical analysis: (**A**) PCA scores plot (CFS group vs. NC group), (**B**) PCA scores plot (Se-TP group vs. CFS group), (**A_1_**) OPLS-DA scores plot (CFS group vs. NC group), (**B_1_**) OPLS-DA scores plot (Se-TP group vs. CFS group).

However, the score plots of the experimental groups were scattered among four quadrants (Figure 6A,B). Therefore, it was necessary to conduct the OPLS-DA analysis, which could make the differences prominent between the interior of the model and the predictive component. As the score plots of OPLS-DA show (Figure 6A_1_,B_1_), the CFS group presented a trend to diverge from the NC group, discovering variation of the metabolic trait in CFS group. This result expressed the changes of some endogenous metabolites due to CFS. These changes shrank after the Se-TP administration, inferring Se-TP’s efficiency against CFS from different aspects.

**Figure 7:**
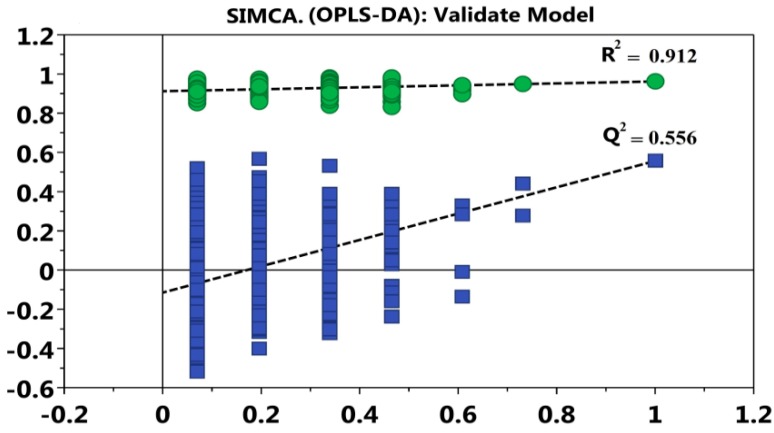
Validation of the OPLS-DA model using a permutation test. The permutations were performed, and the resulting R^2^ and Q^2^ values were plotted. Green circle: R^2^; blue square: Q^2^. The green line represents the regression line for R^2^ and the blue line for Q^2^.

Additionally, the validity of the OPLS-DA model was evaluated using a permutation test. The value of R^2^ represented the interpretability of samples and Q^2^ represented the predictability of the model. Generally, the values of both being more than 0.5 were considered to mean that the OPLS-DA model was valid [24]. As shown in Figure 7, the value of R^2^ was 0.912 and the value of Q^2^ was 0.556, which confirmed that OPLS-DA model was valid in the present research.

In order to filter out irrelevant orthogonal signals and obtain the more reliable differential metabolites, not only variable importance in the projection (VIP) value of metabolite must exceed 1 in the OPLS-DA model but also its *p*-value in the Student’s *t*-test must be less than 0.05 in comparison between groups. KEGG mass spectral libraries were utilized to ensure the identification accuracy of the differential metabolites, and then the similarity value of the compounds needed to exceed 700 to be adopted [24]. After the above filtrations, 11 metabolites were obtained in the CFS group when compared with the NC group; they were malonic acid, fumaric acid, 2-methylfumarate, l-malic acid, 4-acetamidobutyric acid, α-ketoglutaric acid, (R,R)-tartaric acid, d-gluconic acid, palmitic acid, sphingosine, and 21-hydroxypregnenolone. Meanwhile, a total of eight metabolites were obtained in the Se-TP group in comparison with CFS group, including benzoic acid, itaconic acid, glutaric acid, 4-acetamidobutyric acid, creatine, 2-hydroxy-3-isopropylbutanedioic acid, l-dopa, and 21-hydroxypregnenolone (Table 3). 

The peak area relative value of the differential metabolites are shown in Table 4 where the results demonstrated that the values of malonic acid, fumaric acid, 2-methylfumarate, l-malic acid, and palmitic acid were significantly increased, whereas the values of 4-acetamidobutyric acid, α-ketoglutaric acid, tartaric acid, gluconic acid, sphingosine, and 21-hydroxypregnenolone were markedly decreased when compared CFS with NC group. In addition, the values of itaconic acid, glutaric acid, 4-acetamidobutyric acid, creatine, 2-hydroxy-3-isopropylbutanedioic acid, l-dopa, and 21-hydroxypregnenolone were significantly increased but only the value of benzoic acid was markedly decreased when compared Se-TP with CFS group.

Then, the selected metabolites were entered into the MetPA database (http://www.metaboanalyst.ca/) to search for the pathways of metabolites. “Pathway analysis”, “Homo sapiens”, “Hypergeometric test”, and “Relative-betweenness centrality” were selected in metabolic mode, species types, characterization analysis, and path topology analysis, respectively, and then 14 metabolic pathways were generated in the CFS vs. the NC groups, and 4 metabolic pathways were demonstrated in the Se-TP vs. CFS groups (shown in Table 5). 

**Figure 8:**
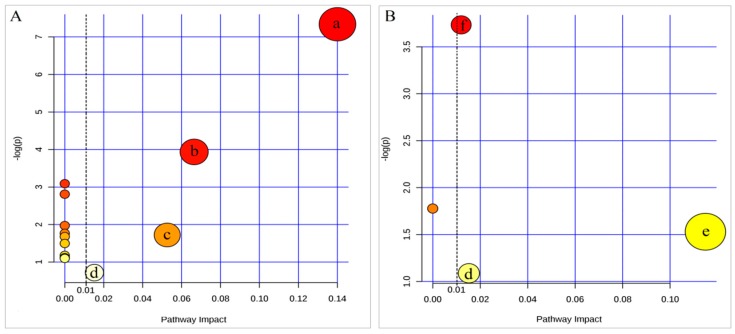
Detection of the metabolic pathway topology analysis. (**A**) CFS group vs. NC group. (**B**) Se-TP group vs. CFS group. (a: citrate cycle; b: alanine–aspartate–glutamate metabolism; c: sphingolipid metabolism; d: steroid hormone biosynthesis; e: tyrosine metabolism; f: arginine-proline metabolism).

Furthermore, the pathway impact values of all differential metabolites were collected via a pathway topology analysis. Four metabolic pathways were found out as disordered pathways in the CFS group when compared with NC (Figure 8A). They were the citrate cycle, alanine-aspartate-glutamate metabolism, sphingolipid metabolism, and steroid hormone biosynthesis. Meanwhile, three metabolic pathways (arginine-proline metabolism, tyrosine metabolism and steroid hormone biosynthesis) were detected in the Se-TP group when compared with CFS (Figure 8B).

## 4. Discussion

Se-TP was obtained from *Ziyang* green tea using the conventional extraction, separation, and purification methods, which was a conjugate with polysaccharide, protein, and selenium. The molecular morphology of polysaccharide was related not only the polymeric degree of molecular but also its biological activity [29]. The previous research found that the surface topography of protein–polysaccharide conjugates usually appeared in a spherical, flocculent, or sheet structure with a diameter of 0.5–10 μm [14,30,31]. The molecular morphology of Se-TP showed many spherical particles with a diameter of 5 μm, but its surface was very smooth. Furthermore, it remains to be further studied whether the form is related to the existence of Se in polysaccharide molecular.

Generally, the mental and physical stimulation were usually simulated and researched in the CFS animal model [32,33,34], and the last stage of a CFS model needs more than two weeks [35,36]. The patients with CFS struggled with decreased memorizing ability, constantly lost interest and attention, and reported disappointed or depressed [37,38,39]. The water maze test is commonly adopted to measure the learning and memory capabilities of small animals [40]. The open-field test is confirmed to measure the anxiety and excitability of the rats. In addition, the mental status and depression degree of animals are detected using the tail-suspension test. Assessing rats’ mental status and physical fatigue and collecting body weight, fodder intake, and water intake to check the growth of the rats, and a total of six evaluation indexes of the CFS group significantly changed (increased or decreased) in comparison with those in the NC group, indicating that the CFS model of rats was successfully built to mimic the chronic physical and mental fatigue. Interestingly, the administration of 200 mg/kg of Se-TP made six evaluation indexes reflect as ideal and opposite changes, so the 200 mg/kg Se-TP group was chosen to conduct the next metabonomic test.

BUN is a metabolic product of protein and amino acid, which is one of blood biochemical parameters related to fatigue. BLA is produced via anaerobic glycolysis, which can be further degraded via the tricarboxylic acid cycle for the production of ATP or removed to other tissue for oxidization or gluconeogenesis [15]. The results indicated that the metabolism extent of the protein or amino acid in CFS rats were higher than those in NC rats. As is already known, CORT and ALD are two of the most important steroid hormones of HPA axis. Generally, the HPA axis can secrete more CORT under physical and mental stress or fatigue. However, the research showed that the level of CORT was lower in CFS patients than normal people [41]. We obtained the same results in the present study in which the high dose Se-TP treatment was demonstrated to improve the levels of BUN and CORT in CFS rats.

The GC-MS method was performed to study the metabolomic characteristics of Se-TP on CFS. The score plots of four groups were not obviously separated in principal component analysis (PAC), so further OPLS-DA analysis were performed, which is a multivariate data analysis with supervision to distinguish the variables of metabolites between groups [42,43]. Comparing with other groups, the OPLS-DA score plots of the Se-TP had an apparent deviation and expressed the changes of some endogenous metabolites due to CFS. The VIP, *p*-value, and similarity were necessary criteria to identify and select the different metabolites. According to above criteria, a total of 11 differential metabolites were filtrated in CFS group; meanwhile, a total of 8 metabolites markedly changed in the Se-TP group. In previous studies about CFS and polysaccharide treatment, we have found five and six different differential metabolites from *Herba Epimedii* polysaccharides and *Schisandra chinensis* polysaccharides treatment, respectively [30,31]. These results indicated that the different polysaccharides improved CFS through the diverse metabolic pathways. Nagyszakal et al. reported that the levels of choline, carnitine, and complex lipid metabolites were changed in CFS patients through plasma metabolomics measurement [44]. Castromarrero et al. demonstrated that the blood-derived extracellular vesicles were significantly induced in CFS patients [45]. These research results may open a new door to identifying novel potential biomarkers in the blood and urine samples of CFS patients.

In order to find CFS’s potential metabolic pathways, the differential metabolites mentioned above were analyzed through MetPA. Yamano and Kataoka reported that the diagnostic biomarker of CFS might come from the immune, endocrine, and metabolic system [46]. In present research, fourteen metabolic pathways were generated in the CFS vs. the NC groups, and four metabolic pathways were generated in the Se-TP vs. CFS groups. Furthermore, the pathway impact values of all differential metabolites were collected via a pathway topology analysis. Three metabolic pathways (citrate cycle, alanine–aspartate–glutamate metabolism, sphingolipid metabolism, and steroid hormone biosynthesis) were found out as disordered pathways because of CFS. Arginine-proline metabolism, tyrosine metabolism, and steroid hormone biosynthesis were found out as disordered metabolic pathways after Se-TP treatment. Since citrate cycle reflect the common route of catabolism of energy substance, such as carbohydrate, fat acid, and protein. Citrate cycle metabolic pathway can reflect the metabolic state of any energy substance in the human body [47]. Alanine, aspartate, and glutamate metabolism play a key role in lymphocyte proliferation and phagocytosis [48]. Sphingosine is the core metabolite in sphingomyelin metabolism and takes part in the generation of ceramide, which improves the learning, memorizing, cognitive and immune abilities [49,50]. 21-Hydroxypregnenolone joins in the steroid hormone biosynthesis, which does well in helping with memorization, anti-fatigue, and refreshing the neural system [51,52]. It is considered that HPA axis abnormality is a common symptom of CFS patients [8]. In general, CFS patients have a lower cortisol ratio in plasma, since HPA axis’ function is disordered [41].

For all the detected metabolites, 4-acetamidobutyric acid and 21-hydroxypregnenolone were joint differential metabolites in the two comparisons (CFS vs. NC and Se-TP vs. CFS). The metabolic pathways of these were attributed to arginine-proline metabolism and steroid hormone biosynthesis, respectively. Pathways could be selected out with a threshold of 0.01, after which the steroid hormone biosynthesis could become the related metabolic pathway illustrating the metabolic mechanism of Se-TP against CFS. It was well known that 21-hydroxypregnenolone acts in a most important role during steroid hormone biosynthesis. Corticosterone and aldosterone were the direct and indirect downstream products based on the analysis of its metabolic pathway. This inference was dramatically in conformity with the lower levels of CORT and ALD in serum. Our findings revealed that the level of 21-hydroxypregnenolone in the CFS rats increased markedly after Se-TP administration. As a result, we inferred that Se-TP administration may make the HPA axis have an abnormal affection for CFS by increasing corticosterone or aldosterone. In addition, our results also implied that the impact values of both tyrosine metabolism and arginine proline metabolism presented a changing trend in pathways after Se-TP treatment. We conclude that the therapeutic mechanisms of Se-TP against CFS were multi-faceted.

## 5. Conclusions

In the present research, a CFS-rats model was established, and Se-TP, which was a selenium–protein–polysaccharide conjugate, was used to treat the rats to evaluate the therapeutic effect on CFS. The result demonstrated that the high dose of Se-TP made effective treatment. The GC-MS method was performed to study the metabolomic characteristics of Se-TP on CFS. Our results showed Se-TP treatment dramatically improved the level of protein or amino acid of CFS rats, and eight differential metabolites were screened out as biomarkers on CFS. Meanwhile, three metabolic pathways were proved to be key improving items after Se-TP treatment according to the above differential metabolites. Therefore, Se-TP administration might improve the function of the HPA axis and immune cells of CFS rats.

## Figures and Tables

**Table 1:** Evaluation results of rats in CFS model.

Groups (*n* = 10)	Body Weight (g)		Diet		Morris Water Maze Test		Open-Field Test		Tail-Suspension Test
	Initial Weight	Last Weight	Fodder (g/d)	Water (mL/d)	Time of Searching for Platform (s)	Number of Times	Number of Times	Number of Standing Events	Motionless Time (s)
NC	149.25 ± 10.63	201.37 ± 9.09	20.84 ± 4.45	54.65 ± 8.97	31.22 ± 14.53	1.38 ± 1.30	46.25 ± 5.23	15.88 ± 4.12	78.75 ± 15.37
CFS	151.41 ± 10.27	177.90 ± 11.13 ^aa^	16.61 ± 3.15 ^aa^	45.89 ± 12.45	50.09 ± 18.09 ^a^	0.75 ± 0.71	33.69 ± 5.19 ^aa^	11.50 ± 2.48 ^a^	100.50 ± 11.94 ^aa^
Se-TP (100 mg/kg)	148.98 ± 9.56	181.38 ± 14.27 ^aa^	17.65 ± 4.17	47.64 ± 13.27	45.66 ± 10.97 ^a^	1.12 ± 0.67	36.58 ± 7.21 ^aa^	14.26 ± 3.85	89.55 ± 9.13 ^b^
Se-TP (200 mg/kg)	149.17 ± 9.81	188.79 ± 9.02 ^aab^	19.11 ± 2.98	43.88 ± 7.09 ^aa^	37.53 ± 5.09 ^b^	1.34 ± 0.97	39.43 ± 6.31 ^ab^	14.28 ± 3.67 ^b^	84.36 ± 11.71 ^bb^

^a^: *p* < 0.05, ^aa^: *p* < 0.01 vs. NC group; ^b^: *p* < 0.05, ^bb^: *p*< 0.01 vs. CFS group.

**Table 2:** Analysis of serum metabolites.

Groups (*n* = 10)	BUN (mmol/L)	BLA (mmol/L)	CORT (ng/mL)	ALD (pg/mL)
NC	6.33 ± 1.82	3.65 ± 0.76	152.49 ± 12.45	72.36 ± 5.26
CFS	8.24 ± 2.05 ^a^	3.77 ± 1.62	141.84 ± 9.37 ^a^	68.48 ± 5.71
Se-TP (100 mg/kg)	6.54 ± 2.26	2.94 ± 0.86	149.73 ± 8.92	73.12 ± 6.53
Se-TP (200 mg/kg)	6.29 ± 1.47 ^b^	3.76 ± 1.41	156.12 ± 16.07 ^b^	67.57 ± 4.85 ^a^

^a^: *p* < 0.05, vs. NC group; ^b^: *p* < 0.05, vs. CFS group.

**Table 3:** Differential metabolites of CFS group vs. Se-TP group.

	No.	RT (min) ^a^	*m*/*z*^b^	KEGG ^c^	VIP	*p*-Value	Metabolite	Trend
CFS vs. NC	1	8.87	188	C00383	2.34	0.008	Malonic acid	↓
	2	11.00	245	C00122	1.87	0.013	Fumaric acid	↑
	3	11.65	184	C01732	1.81	0.020	2-methylfumarate	↑
	4	12.73	233	C00149	2.05	0.020	l-malic acid	↑
	5	12.93	122	C02946	1.67	0.044	4-acetamidobutyric acid	↓
	6	13.83	304	C00026	1.03	0.034	α-ketoglutaric acid	↓
	7	14.48	292	C00898	2.390	0.002	(R,R)-tartaric acid	↓
	8	18.27	333	C00257	2.018	0.018	d-gluconic acid	↓
	9	18.77	117	C00249	1.92	0.013	Palmitic acid	↑
	10	22.16	204	C00319	2.16	0.021	Sphingosine	↓
	11	26.57	73	C05485	2.82	0.000	21-hydroxypregnenolone	↓
Se-TP vs. CFS	1	9.74	179	C00180	1.33	0.045	Benzoic acid	↓
	2	10.88	215	C00490	1.63	0.015	Itaconic acid	↑
	3	11.71	97	C00489	1.71	0.026	Glutaric acid	↑
	4	12.93	122	C02946	2.02	0.027	4-acetamidobutyric acid	↑
	5	13.55	115	C00300	1.49	0.043	Creatine	↑
	6	13.77	247	C04411	1.78	0.041	2-hydroxy-3-isopropylbutanedioic acid	↑
	7	18.90	268	C00355	1.80	0.018	l-dopa	↑
	8	26.57	73	C05485	1.72	0.035	21-hydroxypregnenolone	↑

a: retention time of chromatographic peak; b: mass-to-charge ratio; c: code in KEGG database; ↑: up-regulated; ↓: down-regulated.

**Table 4:** The peak area relative value of the differential metabolites.

R.T.	Metabolites	NC Group	CFS Group	Se-TP Group
8.87	Malonic acid	0.0029 ± 0.0022	0.0005 ± 0.0001 ^aa^	0.0007 ± 0.0003 ^aa^
9.74	Benzoic acid	0.0412 ± 0.0169	0.0433 ± 0.0056	0.0327 ± 0.0003 ^bb^
10.88	Itaconic acid	0.0078 ± 0.0027	0.0059 ± 0.0045	0.0098 ± 0.0036 ^b^
11.00	Fumaric acid	0.0412 ± 0.0189	0.0757 ± 0.0387 ^a^	0.1059 ± 0.1363
11.65	2-methylfumarate	0.0059 ± 0.0015	0.0075 ± 0.0015 ^a^	0.0094 ± 0.0032 ^aa^
11.71	Glutaric acid	0.0105 ± 0.0067	0.0103 ± 0.0057	0.0311 ± 0.0229 ^a,b^
12.73	l-malic acid	0.1060 ± 0.0473	0.2084 ± 0.1295 ^a^	0.2213 ± 0.1680
12.93	4-acetamidobutyric acid	0.0016 ± 0.0007	0.0011 ± 0.0001 ^a^	0.0015 ± 0.0014 ^b^
13.55	Creatine	0.5819 ± 0.5568	0.8729 ± 0.4223	1.3787 ± 0.4483 ^aa,b^
13.77	2-hydroxy-3-isopropylbutanedioic acid	0.0227 ± 0.0124	0.0306 ± 0.0189	0.0735 ± 0.0472 ^aa,b^
13.83	α-ketoglutaric acid	0.0009 ± 0.0004	0.0005 ± 0.0002 ^a^	0.0007 ± 0.0003
14.48	Tartaric acid	0.0035 ± 0.0022	0.0013 ± 0.0002 ^aa^	0.0017 ± 0.0008 ^a^
18.27	Gluconic acid	0.0026 ± 0.0012	0.0017 ± 0.0002 ^a^	0.0018 ± 0.0003
18.77	Palmitic acid	0.0273 ± 0.0076	0.0393 ± 0.0160 ^a^	0.0340 ± 0.0084
18.90	l-dopa	0.0057 ± 0.0021	0.0042 ± 0.0017	0.0062 ± 0.0023 ^b^
22.16	Sphingosine	0.0023 ± 0.0004	0.0014 ± 0.0012 ^a^	0.0021 ± 0.0020
26.57	21-hydroxypregnenolone	0.0022 ± 0.0003	0.0016 ± 0.0008 ^a^	0.0027 ± 0.0011 ^b^

^a^: *p* < 0.05, ^aa^: *p* < 0.01 vs. NC group. ^b^: *p* < 0.05, ^bb^: *p* < 0.01 vs. CFS group.

**Table 5:** Comparative results for the related metabolic pathways in CFS vs. NC and Se-TP vs. CFS.

	Pathway Name	Total ^a^	Hits ^b^	*p* ^c^	−log(*p*)	Holm *p* ^d^	FDR ^e^	Impact Score ^f^
CFS vs. NC	Citrate cycle	20	3	0.001	7.340	0.053	0.053	0.140
	Alanine-aspartate-glutamate metabolism	24	2	0.019	3.936	1.0	0.791	0.066
	d-Glutamine and d-glutamate metabolism	5	1	0.046	3.088	1.0	1.0	0.0
	Arginine-proline metabolism	44	2	0.060	2.808	1.0	1.0	0.0
	Glyoxylate-dicarboxylate metabolism	16	1	0.139	1.972	1.0	1.0	0.0
	Butanoate metabolism	20	1	0.171	1.766	1.0	1.0	0.0
	Sphingolipid metabolism	21	1	0.179	1.721	1.0	1.0	0.053
	Pyruvate metabolism	22	1	0.187	1.679	1.0	1.0	0.0
	Fatty acid elongation in mitochondria	27	1	0.224	1.495	1.0	1.0	0.0
	Fatty acid metabolism	39	1	0.308	1.177	1.0	1.0	0.0
	Tyrosine metabolism	42	1	0.328	1.116	1.0	1.0	0.0
	Biosynthesis of unsaturated fatty acids	42	1	0.328	1.116	1.0	1.0	0.0
	Fatty acid biosynthesis	43	1	0.334	1.096	1.0	1.0	0.0
	Steroid hormone biosynthesis	70	1	0.488	0.718	1.0	1.0	0.015
Se-TP vs. CFS	Arginine-proline metabolism	44	2	0.024	3.734	1.0	1.0	0.012
	Glycine-serine-threonine metabolism	32	1	0.169	1.778	1.0	1.0	0.0
	Tyrosine metabolism	42	1	0.216	1.530	1.0	1.0	0.115
	Steroid hormone biosynthesis	70	1	0.337	1.088	1.0	1.0	0.015

^a^ Total number of metabolites in the pathway; ^b^: Number of the changed metabolites in the pathway; ^c^: *p*-value after enrichment analysis of the metabolic pathway; ^d^: *p*-value after multiple hypothesis testing calibration using Holm–Bonferroni method; ^e^: False discovery rate; ^f^: Threshold of the impact score in topology analysis > 0.01.

## Supplementary Materials

The following are available online at http://www.mdpi.com/2073-4360/10/11/1269/s1, Figure S1: Total ion chromatograms (TICs) of the urine samples obtained from the NC (A), CFS (B), and Se-TP groups (C).
