# Supplementary material for: Therapeutic Effect and Metabolic Mechanism of A Selenium-Polysaccharide from Ziyang Green Tea on Chronic Fatigue Syndrome
Source: Polymers (Basel). 2018 Nov 15;10(11):1269. doi: 10.3390/polym10111269 (PMC6401680; doi:10.3390/polym10111269)
Supplement: Supplementary file 1 [file polymers-10-01269-s001.pdf]

Supplementary Materials:

Therapeutic Effect and Metabolic Mechanism of A Selenium-Polysaccharide from *Ziyang* Green Tea on Chronic Fatigue Syndrome

Chang-zhuan Shao [1,2], Jing Song [2], Shan-guang Zhao [2], Hong-ke Jiang [1], Bao-ping Wang [2] and Ai-ping Chi [2,*]

1 College of Arts and Sciences, Shanghai Maritime University, Shanghai 201306, China; czshao@shmtu.edu.cn (C.S); hkjiang@shmtu.edu.cn (H.J.)

2 Laboratory of Nutrition and Hygiene, Shaanxi Normal University, Xi'an 710119, China; BSUjingjing@163.com (J.S.); zhaoshan@snnu.edu.cn (S.Z.); wbp1979@snnu.edu.cn (B.W.)

* Correspondence: chimu@snnu.edu.cn; Tel.: +86-029-85310156

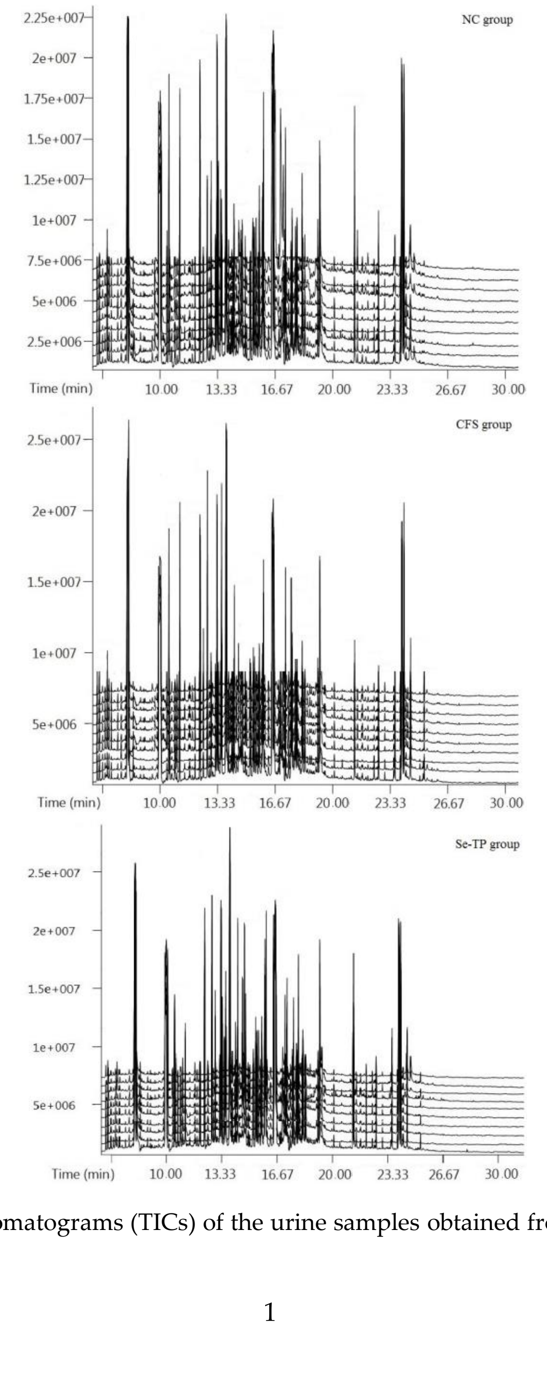

Figure S1. Total ion chromatograms (TICs) of the urine samples obtained from the NC (**A**), CFS (**B**), and Se-TP groups (**C**).
